# High S/N Ratio Slotted Step Piezoresistive Microcantilever Designs for Biosensors

**DOI:** 10.3390/s130404088

**Published:** 2013-03-26

**Authors:** Mohd Zahid Ansari, Chongdu Cho

**Affiliations:** Department of Mechanical Engineering, Inha University, 253 Yonghyun-dong, Nam-Ku, Incheon 402-751, Korea; E-Mail: ansari.zahid@hotmail.com

**Keywords:** step microcantilever, self-heating, biosensors, piezoresistivity, thermal drifting, bimetallic effect

## Abstract

This study proposes new microcantilever designs in slotted step configuration to improve the S/N ratio of surface stress-based sensors used in physical, chemical, biochemical and biosensor applications. The cantilevers are made of silicon dioxide with a u-shaped silicon piezoresistor in p-doped. The cantilever step length and piezoresistor length is varied along with the operating voltage to characterise the surface stress sensitivity and thermal drifting sensitivity of the cantilevers when used as immunosensor. The numerical analysis is performed using ANSYS Multiphysics. Results show the surface stress sensitivity and the S/N ratio of the slotted step cantilevers is improved by more than 32% and 22%, respectively, over its monolithic counterparts.

## 1. Introduction

Piezoresistive microcantilevers sensors have been successfully used in numerous high sensitivity demanding applications such as atomic force microscopy [1–4], force sensor [5,6], DNA sequencing [7], vapour sensor [8] and biosensors [9–11]. The integrated readout ability of these sensors makes them especially useful. The other advantages include compact size and low power consumption features that make them very convenient to conduct out-of-lab studies. In addition, the sensor can also be operated in optically obscure fluids. Nevertheless, the piezoresistive microcantilever sensors have a drawback in form of lower sensitivity when compared to their optical readout counterparts. The sensitivity is reduced because of electrical sources of noise such as Johnson noise and Hooge noise (or 1/f noise) [12,13], and mechanical ones like thermal drifting noise and excitations [12–15]. Johnson noise is produced because of temperature-dependent random motion of mobile charge carriers in the piezoresistor, whereas, Hooge noise is because of fluctuating electrical conductivity of the piezoresistor. Thermal drifting occurs because of operating temperature conditions and the self-heating in the piezoresistor when voltages are applied.

The sensitivity of piezoresistive microcantilever sensors can be improved by improving its signal-to-noise (S/N) ratio by means of modifying the cantilever design as well as by reducing or eliminating noise from the signals measured. The present study focuses on cantilever design modifications. The sensitivity of piezoresistive microcantilevers depends on converting the surface stress into higher resistance change in their piezoresistor. This can be achieved by increasing the cantilever tip deflections and therefore the resulting normal stresses that are induced in the piezoresistor. In addition, for maximum conversion the piezoresistor should be placed strategically in the high stress zones in the cantilever which normally occur near the fixed end and close to the top/bottom surface. The high deflection and high stress requirements can be fulfilled by changing the cantilever profile [16–18], geometry [19–22], surface modification [23,24] and material [25–28]. In addition, the properties of the piezoresistor and operating conditions can also be very influential on its sensitivity [12–14, 29 and 30].

In this study, the authors propose new slotted step cantilever designs that can be used as piezoresistive microcantilever sensor to improve its sensitivity and S/N characteristics. The cantilevers are made of silicon dioxide with a u-shaped silicon piezoresistor doped with boron. The step length and the piezoresistor length are varied in the designs. In total, six slotted step microcantilever designs are studied here and their performance is compared. A commercial finite element analysis software ANSYS Multiphysics is utilized to characterize the performance of the proposed designs when used as the sensing element in an immunosensor to study analyte-receptor binding reaction. In addition, the designs are subjected to bias voltages in order to characterize their thermal drifting vulnerability during operation. Finally, the sensitivities of the designs to the surface stress and to the thermal drifting and their S/N characteristics are discussed.

## 2. Theory and Modelling

Surface stresses are stresses that occur at and are confined only to the surface of a solid, usually within few atomic planes from the surface. These stresses are produced mainly by the adsorption of foreign atoms onto the surface or the redistribution of the electronic charge at the surface due to the change in the equilibrium positions of the atoms near the surface [31]. The stresses can be tensile or compressive. The adsorbate-induced surface stress variations provide a very convenient and high sensitive means to study analyte-receptor type chemical, biochemical and biological binding interactions using microcantilever sensors.

**Figure 1.**
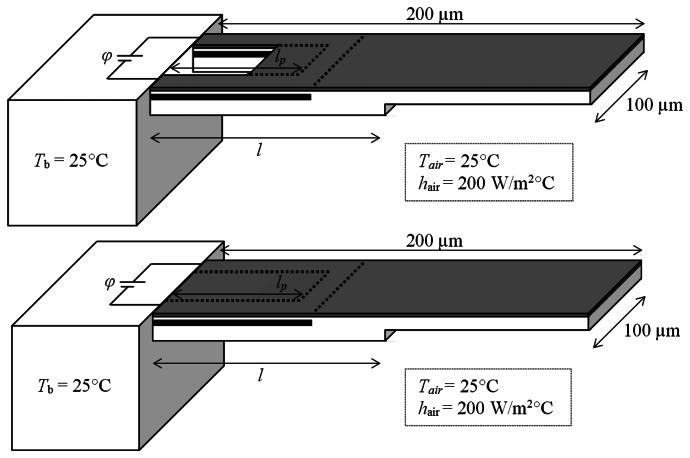
Schematic of slotted (**top**) and monolithic (**below**) step microcantilever designs with u-shaped piezoresistor.

In case of piezoresistive microcantilever sensors, the adsorbate-induced surface stress change on the functionalised surface of the cantilever induces stress in its piezoresistor and changes its electrical resistance. The measurement of the change in resistance provides the information about the type and concentration of the adsorbing analyte. Figure 1 shows the schematic diagrams of the step piezoresistive microcantilever in slotted and monolithic configurations. The fixed-end thickness is 1 µm and the free-end thickness is 0.5 µm. In the slotted designs, a rectangular hole of length 50 µm and width 60 µm is introduced abutting the fixed end. The cantilevers are made of silicon dioxide with a u-shape silicon piezoresistor with p-type dopant. The width of piezoresistor is constant at 20 µm. A thin film of gold is also deposited on the top surface to help monolayer assembly of analyte molecules. The analyte-receptor interactions are assumed occur at the top surface and produce tensile surface stress.

Piezoresistivity is the property of a material to change its electrical resistance when stresses occur. This effect is more common in semiconductors and depends on numerous factors including dopant type and its concentration. The relationship between the resistance change (Δ*R*/*R*_0_) and the stress along the longitudinal axis of the crystal (*σ*_l_) can be given as Δ*R*/*R*_0_ = *π*_l_*σ*_l_, where *π*_l_ is longitudinal coefficient of piezoresistivity. Thus, the surface stress sensitivity of a piezoresistive microcantilever sensor can be defined as:

(1) $$S_{S}={\left(\frac{\Delta R}{R_{0}}\right)}_{S}=\pi_{l}\sigma_{l,S}$$

Here, *σ*_l,S_ is the longitudinal component of the stress induced in the piezoresistor because of the applied surface stress. In this study, we assumed the piezoresistor is p-doped and *π*_l_ = 72 × 10^−11^ Pa^−1^ [32]. Zhou *et al.* [11] reported that a surface stress of about 0.04 N/m is produced in their piezoresistive microcantilever immunosensor during antigen–antibody interaction of Immunoglobulin G (IgG) at a concentration of about 2,000 ng/mL. We will use this surface stress value to study the performance of the cantilever designs shown in Figure 1 when used as immunosensor.

Self-heating and the consequent thermal drifting is a source of inaccuracy in case of piezoresistive microcantilevers. The electric current passing through the piezoresistor generates self-heating which increases the operating temperature of the microcantilever. The increase in temperature produced thermal drifting in the cantilevers in form of bimetallic bending in the cantilever and variations in the original resistance/piezoresistance properties of the doped piezoresistor. Bimetallic bending occurs because of the different coefficients of thermal expansion of the constituent layers of the cantilever. In contrast, the electrical resistivity and piezoresistivity are inherently strong function of temperature and their temperature dependency is given by temperature coefficients TCR and TCP, respectively. Thus, the thermal drift sensitivity can be given as:

(2) $$S_{T}={\left(\frac{\Delta R}{R_{0}}\right)}_{T}=\eta \Delta T+\pi_{l}(1+\beta \Delta T)\sigma_{l,T}$$

where, *η* is TCR, *β* is TCP and Δ*T* is the temperature increase in the piezoresistor of the microcantilever. Here, *σ*_l,T_ is the longitudinal component of the total stress induced in the piezoresistor because of bimetallic bending. It should be noted that the temperature coefficients *η* and *β* can be positive or negative depending on the dopant type and its concentration. In the present study, we assumed the piezoresistor is made of p-doped silicon with boron doping concentration about 1 × 10^19^/cm^−3^. For this dopant type and concentration, the TCR and TCP values are about 1 × 10^−4^/°C and −2.7 × 10^−4^/°C, respectively [33]. Since the increase in temperature will introduce error in the measurements, the thermal drifting in the piezoresistive microcantilevers should also be determined to characterise their thermal vulnerability.

## 3. Numerical Analysis

**Figure 2.**
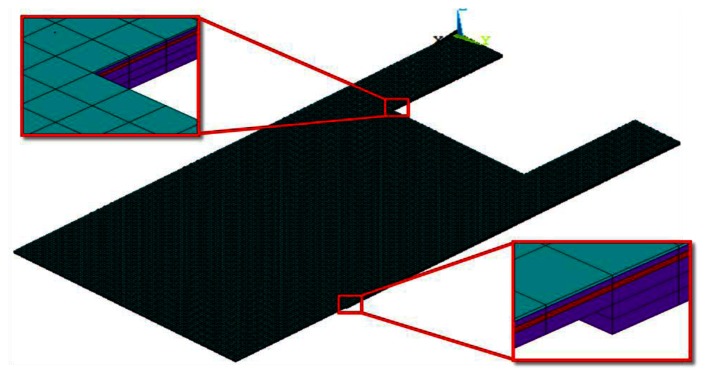
Finite element model of the slotted microcantilever showing the piezoresistor and substrate layout details (inset).

The numerical analysis was conducted using a commercial finite element analysis software ANSYS Multiphysics v.13. The finite element models were meshed using scalar SOLID5 elements and were solved under steady state conditions. These elements can accurately simulate the multi-field physics problems involving scalar quantities like temperature and voltage. The solution convergence test was carried for a number to cases to minimise computational error. In total, about 82,000 and 92,000 elements were used for the two step lengths of 100 µm and 150 µm, respectively. The geometric properties and the ambient operating conditions of slotted and monolithic microcantilevers are presented before in Figure 1. The top-down thickness of the constituent layers was 0.05 µm (Au film), 0.1 µm (SiO_2_ insulation), 0.1 µm (p-doped Si piezoresistor), 0.25 µm (SiO_2_ insulation) and 0.5 µm (SiO_2_ substrate step). The finite element model showing the detailed layered structure of the slotted cantilever is shown in Figure 2. The piezoresistor element is indicated in red. The material properties of the layers are listed in Table 1.

The numerical studies included surface stress, modal and thermo-electric analysis of the slotted and monolithic step piezoresistive microcantilever designs shown in Figure 1. The step length (*l*) was changed as 100 µm and 150 µm. And, the piezoresistor length (*l*_p_) was changed as (i) wrapped around the rectangular slot (*i.e.*, *l*_p_ = 50 µm), (ii) overlap the cantilever step (*i.e.*, *l*_p_ = *l*) and (iii) cover the entire length (*i.e.*, *l*_p_ = 180 µm) of the cantilever to find the most suitable design for the piezoresistor to be created inside the microcantilever. Thus, three different piezoresistor length variations for each step length and therefore in total six different (*l*, *l*_p_) designs were studied. The results were also compared against their monolithic ones. The bias voltage (*φ*) was increased from 0 V to 5 V. The initial cantilever temperature was 25 °C.

In the first analysis, a surface stress of 0.04 N/m was applied to the top surface of the cantilevers and the results for maximum deflection and maximum von Mises stress in the microcantilevers were obtained. The surface stress was modelled as in-plane, bidirectional tensile force acting along the top surface. In the second study, modal analysis was performed to determine the fundamental resonant frequency of the designs. In the third and final analysis, thermo-electric simulation was performed to study the self-heating and the thermal drifting associated with the designs when bias voltages are applied. The convective heat loss boundary conditions were applied at the top and bottom surfaces of the cantilevers only. The heat loss from the edges of the cantilever was neglected. In all the cases, the results for average longitudinal stress (*σ*_l_) and the average temperature increase (Δ*T*) in the piezoresistor were determined. The results were then used to calculate the surface stress sensitivity and thermal drift sensitivity by using Equations (1) and (2), respectively; and finally to determine the S/N characteristics.

## 4. Results and Discussion

Table 2 presents the numerical analysis results of different (*l*, *l*_p_) microcantilevers for the maximum surface stress-induced cantilever tip deflection (Δ*z*), the fundamental resonant frequency (*f*_0_) and the maximum von Mises stress (*σ*). The results are normalised by their respective values for the monolithic designs. The applied surface stress was 0.04 N/m. This can be seen in the table that the change in step design from monolithic to slotted configuration increased the deflection and the stress values but decreased the frequency. The change in piezoresistor length from *l*_p_ = 100 µm to *l*_p_ = 180 µm, however, has negligible effect on these quantities. The high deflections can be attributed to the reduced bending stiffness of the cantilevers because of the rectangular slot at their fixed ends (Figure 1). The reduction in bending stiffness is also responsible for the reduction in the resonant frequency of the slotted cantilevers compared to the monolithic. Based on the results presented in Table 2, we have concluded that slotted step designs have better deflection and stress properties than monolithic ones and will produce larger resistance change in the piezoresistive microcantilevers.

**Figure 3.**
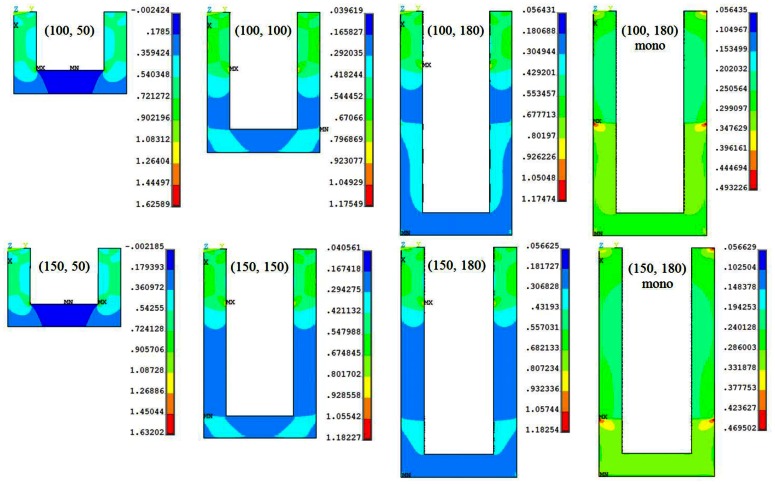
The longitudinal stress distribution (MPa) in different (*l*, *l*_p_) piezoresistor designs for surface stress 0.04 N/m.

The surface stress-induced longitudinal stress (*σ*_l,S_) distribution in the piezoresistor elements of the slotted piezoresistive microcantilever designs in shown in Figure 3. The top edge is the fixed end of the cantilever and is fully constrained. The results for monolithic designs are also presented. The maximum stress in slotted designs is more than twice compared to the monolithic. For a given piezoresistor length, the figures show the stress magnitude is very similar in both the step lengths. In contrast, this is also evident in Figure 3 that the change in piezoresistor length has significant effect on the stress magnitude and its distribution in the cantilevers, especially in case of (100, 50) and (150, 50) designs. The maximum stress in these designs is about 35% higher. Nevertheless, the resonant frequency of these designs is also the lowest (see Table 2). Thus, in case the high frequency requirement is not critical, (100, 50) and (150, 50) are the most suitable designs to achieve high stress and therefore high resistance change in piezoresistive microcantilevers.

**Figure 4.**
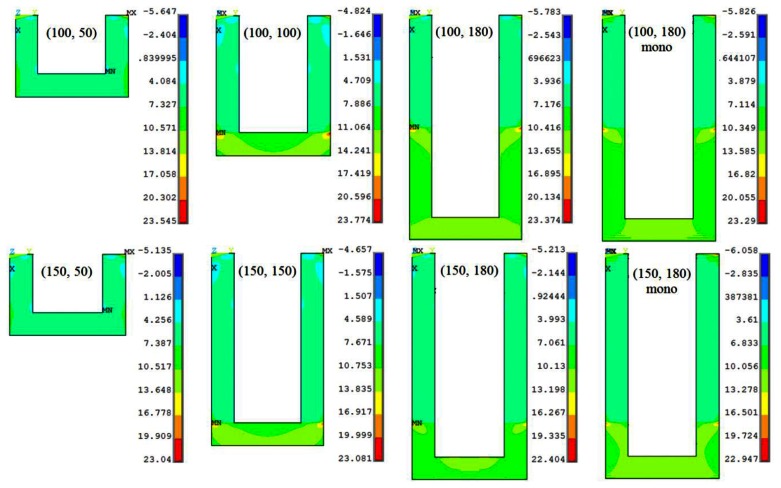
The longitudinal thermal stress distribution (MPa) in different (*l*, *l*_p_) microcantilever designs for bias voltage 5 V.

Figure 4 shows the self-heating-induced longitudinal thermal stress (*σ*_l,T_) distribution in the piezoresistor of slotted and monolithic cantilevers subjected to a bias voltage of 5 V. The top edge is the fixed end of the cantilever where the bias voltages are applied. It is interesting to note that the maximum thermal stress in all the cantilevers is nearly same in magnitude. In other words, the change in the piezoresistor or the step length has negligible effect on the maximum thermal stress in the cantilevers. This is in contrast to the mechanical stress values shown in Figure 2 wherein the stress values for (100, 50) and (150, 50) designs are the highest for the two step lengths. Another observation in Figure 4 is that the values of thermal stress are much larger than mechanical stress shown in Figure 3. This means the resistance change produced by the self-heating effect in form of bimetallic bending will be much larger than that by the surface stress-induced bending (*i.e.*, signal) and therefore will produce large noise in the measurements.

Another major contributor to the noise is the change in resistance properties of the piezoresistor because of its temperature coefficients TCR/TCP. To characterize this effect, the temperature variation study was performed. Table 3 presents the average temperature increase (Δ*T*) in the piezoresistor element of the slotted and monolithic step cantilevers when voltages are applied. The initial piezoresistor temperature was 25 °C. It is obvious in the table that for a given cantilever model the temperature increases about quadratically with the applied voltages. This observation is consistent with the analytical models of temperature increase presented by the authors in [15,29]. This can also be observed that the increase in step length has negligible effect on the temperature increase in the piezoresistor. However, the values decrease with the increases in the piezoresistor length. This can be attributed to the relatively larger surface area of the cantilever section containing the piezoresistor exposed to convective heat loss to the ambient. As the piezoresistor length is increased the length of the cantilever section containing it is also increased. The larger the surface area, the higher the heat loss will be.

**Figure 5.**
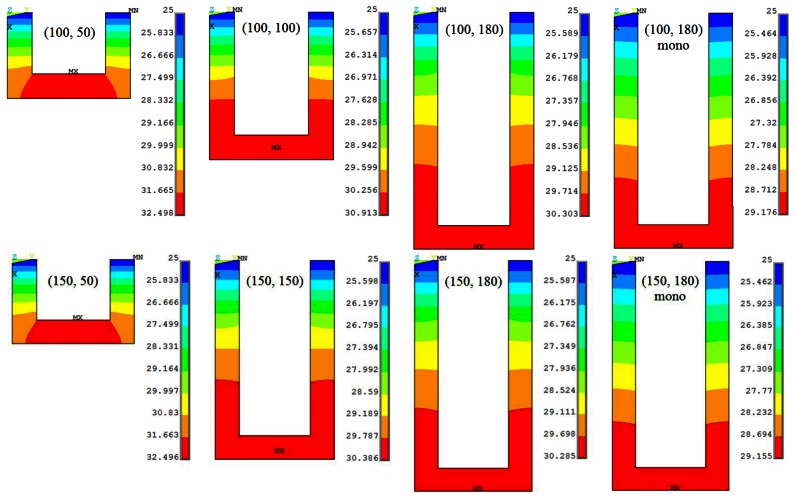
The temperature distribution (°C) in different (*l*, *l*_p_) piezoresistor designs for bias voltage 5 V.

It is interesting to note that though the piezoresistor length is same the monolithic designs show less temperature increase than their slotted counterpart. This behaviour can be explained by the less thermal mass capacity of the slotted cantilevers. The removal of material from the cantilever in order to make the rectangular slot will reduce its thermal mass capacity, and therefore will result in high temperature rise for the given amount of self-heating. Thus, we can conclude that cantilevers with short piezoresistor are more vulnerable to TCR/TCP effects. The self-heating-induced temperature distribution in the piezoresistor the cantilevers subjected to a bias voltage of 5 V is shown in Figure 5.

As mentioned before, Johnson noise and Hooge noise are the main electrical sources of noise in piezoresistive microcantilever sensors. Johnson noise is produced because of the thermally charged random motion of the carriers in the piezoresistor. The Johnson power noise spectral density *S*_VJ_ for a resistance *R* at temperature *T* is defined as *S*_VJ_ = 4*k*_B_*TR*, where *k*_B_ is the Boltzmann constant [13]. Since the temperature increase is more significant in case of cantilevers with short piezoresistor, see Table 3, it appears that the Johnson noise will present significant problem in such microcantilever designs. However, it should also be noted that since resistances increase linearly with the conductor length, a longer piezoresistor will also have higher resistance value. Thus, we see a competing relation between temperature and resistance when the piezoresistor length is increased. The dominant factor can be predicted by analysing their relative effect by using the Johnson noise relation and Table 3.

For instance, changing the cantilever design from (100, 50) to (100, 100) increases the mean total current carrying piezoresistor length (2*l*_p_ + *W*), and therefore the resistance, by about 50%, but the temperature increase is less than 20% for all the voltages applied. By carrying a similar analysis on all the other cantilever designs, we found the resistance is indeed the dominant factor in the cantilever designs to affect the Johnson noise, and for reducing it short piezoresistor should be used. In addition, operating the microcantilevers at lower bias voltage can also be helpful in this regard. Hooge noise is produced because of fluctuating electrical conductivity of the piezoresistor. The Hooge spectral power noise density is given as *S*_VH_ = *αV*^2^/*f N*, where *α* is a parameter, *V* is bias voltage, *f* is frequency and *N* is the total number of carriers [13]. Thus, based on this relation we can say that by using a long piezoresistor, which will increase *N*, operating the cantilever at high frequency and at very low bias voltage can help reduce the Hooge noise.

Table 4 lists the normalised sensitivity results of proposed slotted step piezoresistive cantilevers when used as the immunosensor operated at different bias voltages. The adsorbtion-induced surface stress was 0.04 N/m. The results for monolithic step design are also presented for a comparison. The results for cases when no bias voltage is applied are also listed. These represent the thermal drifting sensitivity of the cantilever designs to the ambient operating environment. It can be clearly observed in the table that the surface stress sensitivity of slotted microcantilever designs is higher than the monolithic. The increase in piezoresistor length, however, decreases the stress sensitivity of slotted designs. It is also evident in the table that the sensitivity of all the piezoresistive microcantilever designs to the surface stress is much lower than to the thermal drifting effects in form of bimetallic deflection and TCR/TCP effects. In addition, the drifting sensitivity increases with the bias voltage, which can be contributed to the high temperature increase and therefore high thermal stresses in the cantilevers. Results suggest the surface stress sensitivity of the slotted cantilevers is less affected by the step length.

Table 5 shows the signal-to-noise ratio (S/N) characteristics of slotted and monolithic step microcantilever designs when biased to different operating voltages. The signal is defined here as the resistance change produced by the application of surface stress only, and the noise is the total resistance change produced by the combined action of the surface stress and thermal drifting. The results are normalised for the two step lengths by their respective monolithic cantilever results. The results show the slotted designs have higher S/N values than their monolithic counterparts. The improvement in S/N values is achieved mainly because of the increased surface stress-induced resistance change in the slotted step microcantilevers (see Table 4). Thus, the increase in signal value resulted in increased S/N values.

It is also evident in Table 5 that the microcantilevers operated at low bias voltages have the highest S/N values. Further, for each step length the slotted cantilever designs with shortest piezoresistor shows the highest values for S/N. Thus, (100, 50) is the best design to be used as the piezoresistive microcantilever-based surface stress measurements. The designs is especially attractive in a sense that it not only have high S/N characteristics but also is less susceptible to Johnson noise and Hooge noise, and should be operated at low voltages to achieve the maximum efficiency. Since operating the sensor at low voltage will also reduce its resolution, appropriate electronic amplifiers can be used to overcome this shortcoming.

The fabrication of monolithic cantilevers can be done using deep reactive-ion etching (DRIE) processes and is already in application [10,11]. The fabrication of slotted step piezoresistive microcantilevers can be achieved by extending the conventional fabrication process for monolithic piezoresistive microcantilevers. The difference between the two is the presence of a step and a rectangular slot at the fixed end. This can be achieved by careful selection of masking material and isotropic/anisotropic etching cycles. It should be noted that because of the layered structure with different material properties, the fabricated slotted step microcantilevers can have significant residual stress which would result in non-zero initial deflection and stress in the cantilevers. Therefore, the development of post-fabrication stress release mechanism is essential to improve the design performance of these cantilevers. The adverse effect of residual stress on cantilever performance can be partly overcome by operating the microcantilevers with a reference microcantilever on the same chip in differential readout along with symmetrical balanced Wheatstone bridge arrangement [3]. The reference microcantilever is not functionalised with receptor molecules and therefore does not participate in analyte-receptor binding reactions. This method is commonly used to eliminate electrical noises and thermal drifting from the total signal and to improve the S/N ratio.

## 5. Conclusions

This study proposed new microcantilever designs to be used as the sensing element in piezoresistive microcantilever sensors based on surface stress variation. In order to demonstrate its efficacy, we used the proposed designs as an immunosensor to study analyte-receptor type binding reaction. The designs were characterised for their surface stress-induced deflections, resonant frequency, surface stress sensitivity, thermal drifting sensitivity and S/N properties. In addition, the Johnson noise and Hooge noise associated with the designs were discussed.

The slotted step designs showed more than 40% increase in surface stress-induced deflection but more than 25% decrease in resonant frequency than their monolithic counterparts. In addition, the increase in step length decreased the deflection, but the frequency results are relatively unchanged. We also found that the increase in piezoresistor length is less influential to surface stress-induced deflection and frequency results. The average piezoresistor temperature increase in the slotted designs decreased with increase in piezoresistor length, but is relatively unchanged when the step length is increased. The piezoresistor temperatures were found to increase nonlinearly with voltage. Microcantilevers with *l*_p_ = 50 µm are best designs to reduce the Johnson noise.

The surface sensitivity of slotted step microcantilevers is more than 32% its monolithic design. However, the thermal drifting sensitivity of these designs was also higher up to 16%. Thus, the slotted designs are more susceptible to thermal drifting. The thermal drifting sensitivity increased nonlinearly with voltage because of high self-heating. The S/N ratios for slotted step microcantilevers were found generally more than 22% over its monolithic design. In addition, S/N ratios were found to increase with decrease in piezoresistor length and step length and the bias voltage. Therefore, for achieving high S/N the slotted step microcantilever with short step length and short piezoresistor length should be selected and operated at low bias voltages. We found the slotted step microcantilever (100, 50) is the best design to be used as the sensing element in a piezoresistive microcantilever sensor.

## Figures and Tables

**Table 1.** Material properties of doped microcantilevers in µMKS units.

**Property**	**SiO_2_**	**Si**	**Au**
Thermal conductivity, *k* (pW/µm °C)	1.38 × 10^6^	150 × 10^6^	317 × 10^6^
Thermal expansion coefficient, *λ* (1/°C)	0.5 × 10^−6^	2.8 × 10^−6^	14.2 × 10^−6^
Specific heat, *c*_p_ (pJ/kg °C)	745 × 10^12^	712 × 10^12^	129 × 10^12^
Electrical resistivity, *ρ*_e_ (Tohm-µm)	-	1 × 10^−9^	-
Elastic modulus, *E* (MPa)	70 × 10^3^	160 × 10^3^	80 × 10^3^
Poisson's ratio, *ν*	0.20	0.23	0.42
Mass density, *ρ* (kg/µm^3^)	2.22 × 10^−15^	2.32 × 10^−15^	19.3 × 10^−15^

**Table 2.** Normalised deflection, frequency and stress results in different (*l*, *l*_p_) microcantilever designs.

**Design**	**Δ*z***	***f*_0_**	***σ***
(100,50)	1.51	0.75	1.15
(100,100)	1.45	0.75	1.04
(100,180)	1.44	0.75	1.03
(100,180) Mono	1	1	1

(150,50)	1.67	0.69	1.16
(150,150)	1.66	0.69	1.04
(150,180)	1.66	0.69	1.04
(150,180) Mono	1	1	1

**Table 3.** Normalised average temperature increase in the piezoresistor of different (*l*, *l*_p_) microcantilever designs due to self-heating.

**Design**	**Δ*T***				

	**1 V**	**2 V**	**3 V**	**4 V**	**5 V**
(100,50)	1.82	1.82	1.82	1.82	1.82
(100,100)	1.55	1.55	1.55	1.55	1.55
(100,180)	1.36	1.36	1.36	1.36	1.36
(100,180) Mono	1	1	1	1	1

(150,50)	1.83	1.83	1.83	1.83	1.83
(150,150)	1.41	1.41	1.41	1.41	1.41
(150,180)	1.36	1.36	1.36	1.36	1.36
(150,180) Mono	1	1	1	1	1

**Table 4.** Normalised surface stress sensitivity (*S*_S_) and thermal drifting sensitivity (*S*_T_) results (%Δ*R*/*R*_0_) for different (*l*, *l*_p_) microcantilever designs.

**Design**	***S*_S_**	***S*_T_**					

		**0 V**	**1 V**	**2 V**	**3 V**	**4 V**	**5 V**
(100,50)	1.59	0.96	0.97	0.99	1.03	1.07	1.12
(100,100)	1.51	1.05	1.06	1.07	1.09	1.13	1.16
(100,180)	1.34	1.00	1.00	1.01	1.03	1.05	1.07
(100,180) Mono	1	1	1	1	1	1	1

(150,50)	1.57	0.99	0.99	1.02	1.05	1.10	1.15
(150,150)	1.37	1.01	1.02	1.03	1.05	1.07	1.09
(150,180)	1.32	1.00	1.00	1.02	1.03	1.05	1.07
(150,180) Mono	1	1	1	1	1	1	1

**Table 5.** Normalised S/N ratio for different (*l*, *l*_p_) microcantilever designs.

**Design**	**S/N**				

	**1 V**	**2 V**	**3 V**	**4 V**	**5 V**
(100,50)	1.5731	1.5421	1.4962	1.4416	1.3841
(100,100)	1.3942	1.3761	1.3489	1.3158	1.2801
(100,180)	1.3108	1.2991	1.2813	1.2594	1.2353
(100,180) Mono	1	1	1	1	1

(150,50)	1.5284	1.4986	1.4545	1.4019	1.3466
(150,150)	1.3212	1.3079	1.2878	1.2631	1.2362
(150,180)	1.2935	1.2820	1.2645	1.2429	1.2192
(150,180) Mono	1	1	1	1	1
